# Impact of a hospital improvement initiative in Bangladesh on patient experiences and satisfaction with services: two cross-sectional studies

**DOI:** 10.1186/1472-6963-11-S2-S10

**Published:** 2011-12-21

**Authors:** Khalid Omer, Anne Cockcroft, Neil Andersson

**Affiliations:** 1 CIET in Pakistan, PO Box 13018, Karachi 75350, Pakistan; 2 CIET Trust Botswana, PO Box 1240, Gaborone, Botswana; 3 Centro de Investigación de Enfermedades Tropicales, Universidad Autónoma de Guerrero, Acapulco, México

## Abstract

**Background:**

The Bangladesh government implemented a pilot Hospital Improvement Initiative (HII) in five hospitals in Sylhet division between 1998 and 2003. This included management and behaviour change training for staff, waste disposal and procurement, and referral arrangements. Two linked cross-sectional surveys in 2000 and 2003 assessed the impact of the HII, assessing both patients' experience and satisfaction and public views and use of the hospitals.

**Methods:**

In each survey we asked 300 consecutive outpatients and a stratified random sample of 300 inpatients in the five hospitals about waiting and consultation time, use of an agent for admission, and satisfaction with privacy, cleanliness, and staff behaviour. The field teams observed cleanliness and privacy arrangements, and visited a sample of households in communities near the hospitals to ask about their opinions and use of the hospital services. Analysis examined changes over time in patients' experience and views. Multivariate analysis took account of other variables potentially associated with the outcomes. Survey managers discussed the survey findings with gender stratified focus groups in each sample community.

**Results:**

Compared with 2000, an outpatient in three of the hospitals in 2003 was more likely to be seen within 10 minutes and for at least five minutes by the doctor, but outpatients were less likely to report receiving all the prescribed medicines from the hospital. In 2003, inpatients were more likely to have secured admission without using an agent. Although patients’ satisfaction with several aspects of care improved, most changes were not statistically significant. Households in 2003 were significantly more likely to rate the hospitals as good than in 2000. Use of the hospitals did not change, except that more households used the medical college hospital for inpatient care in 2003. Focus groups confirmed criticisms of services and suggested improvements.

**Conclusion:**

Improvements in some aspects of patients' experience may have been due to the programme, but the decreased availability of medicines in government facilities across the country over the period also occurred in these hospitals. Monitoring patients’ experience and satisfaction as well as public views and use of hospital services is feasible and useful for assessing service interventions.

## Background

The Government of Bangladesh implemented a five year Health and Population Sector Programme (HPSP) from 1998-2003 [1], aiming to improve health services. Despite expansion of health infrastructure and some improvements in health indicators since 1971 [2], in 1998 health services indicators in Bangladesh were poor [3]. Only 45% of the population were covered for essential health care in 1995, against a target of 80% set in 1990 [4]. A 1997 report noted some improvement in access to health services but persisting poor quality of the services [5]. A national survey conducted in early 1999 found only 13% of households used government health services for treatment of recent illness, while 32% used private services; only 37% of households had a good opinion of government health services; 53% of services users rated the service received as good [6]. Outpatient attendance in public hospitals fell from 23.50 million in 1993 to 15.65 million in 1996, with low bed occupancy in secondary care facilities but almost 100% bed occupancy in tertiary hospitals [7,8]. A study in the capital, Dhaka, concluded quality perceptions were driving patients away from public hospitals to private hospitals [9].

The HPSP aimed to improve health services especially among women, children and the poor, through client-centred provision of an Essential Services Package (ESP), mainly of preventive services, plus selected treatment services. One component was intended to improve the management and quality of hospital services. A pilot Hospital Improvement Initiative (HII) began in 1998 in four district hospitals in Sylhet division and the Sylhet medical college hospital [10]. The HII programme in the five pilot hospitals had several different elements. Formal training sessions aimed to build management skills for hospital board members and managers. Other sessions for all hospital staff with patient contact aimed to help them to improve their interaction with patients, giving them more time and explanations, and respecting their privacy. A new system was introduced for waste disposal, separating clinical and non-clinical waste, and staff were trained in the use of the system. Some structural changes were made in the hospitals to improve privacy (such as construction of separate toilets for women) and equipment such as screens was provided. Systems for procurement were reviewed and updated, and relevant staff were trained in the new systems. Part of the effect of this should have been to improve availability of medicines. The programme initially intended also to improve the referral system, reducing self-referrals and use of these tertiary facilities for primary care. In practice, this element did not happen. The HII programme set up a Health Advisory Committee for each pilot hospital, including community representatives, with the intention of integrating the “citizens’ voice” into hospital management.

The HII commissioned a baseline survey in 2000 and a repeat survey in 2003. Designed in consultation with the HII programme team, the aim of the surveys was to evaluate the impact of the HII programme interventions on relevant aspects of patient experiences and satisfaction with services from the hospitals, by collecting data from hospital patients and also from households in communities served by the hospitals. The decision to evaluate the HII programme from the point of view of patients reflected an underlying aim of the programme to make services more client-centred. The surveys were not intended to evaluate the processes of the HII programme; they focused on the experience and views of patients and households at two time points. We shared the findings of the baseline survey in 2000 with local stakeholders, including the management teams of the pilot hospitals and the team implementing the HII programme, and in 2003 we shared the findings from the follow-up survey with the same stakeholders. We prepared technical reports on both surveys [11,12].

The two surveys used the social audit approach. This approach collects evidence (quantitative and qualitative) about services from the people that are meant to benefit from them, and puts this together with evidence from the services themselves, eventually with a view to improving services and helping communities to determine their own health and well being. The philosophy and methods of social audit are described in detail elsewhere [13,14]. The surveys measured the experience and satisfaction of patients visiting the hospitals and documented use of the hospitals and public views about them.

Research on patients’ satisfaction dates from the late 1960s [15]. During the 1990s patients’ assessment of quality of care became prominent, especially in industrialized countries, and patients' satisfaction with health services became an important area of inquiry [16-18]. However, satisfaction surveys have been criticised for being methodologically weak and focussing on the agenda of clinicians and managers rather than patients [19]. Systematic measurement of patients’ experiences may be a more sensitive indicator of empowerment [20]. Researchers in the USA developed and tested a standardised instrument to measure patients’ concerns and experience. The tool was used in the USA in telephone interviews with hospital inpatients and relatives [21], and since then it has been used in Australia, Canada [22] and various European countries [23,24]. In many developed countries, assessing client satisfaction has been institutionalized as a tool for quality assessment of hospital performance and is used frequently at national and sub-national levels [25,26]. There is an extensive literature using patient or client satisfaction with medical services as a measure of quality [27,28]. However, few studies have made international comparisons of hospital performance [29]. Since 2000, international agencies such as the World Health Organization (WHO) and the Organization for Economic Cooperation and Development (OECD) have stressed patient outcomes as a key indicator of system performance [30,31]. In 2000, WHO included an index of responsiveness to the expectations of consumers in its report on health systems around the world [32]. In 2003, the Health Evidence Network report included consumer surveys among four standard methods of measuring hospital performance [33]. Around the same time, the WHO regional office for Europe initiated a project to provide member states with a practical tool to monitor quality of hospital services [34,35]. The tool was piloted in 55 hospitals, all in developed countries [36]. In developing countries, use of patients’ satisfaction as a measure of quality of health services remains relatively rare. Some studies have used patient satisfaction as an indicator of quality of services and some have examined the association between perceived quality of care and health seeking behaviour of patients [9,37-39]. We are not aware of any studies measuring both patient experiences and satisfaction and public views and use of services in order to assess the quality of hospital services.

In this paper we use data from our two surveys to examine formally the changes, over the period of the HII programme, in aspects of patients' experience and satisfaction with hospital services relevant to the elements of service addressed by the programme, and in household views and use of the hospitals, as a way of assessing the potential impact of the programme on these outcomes.

## Methods

### Sample

We selected a household sample and outpatient and inpatient samples for each of the five pilot hospitals: Sylhet Medical College Hospital (MCH), Sylhet District Hospital (DH), Sonamganj DH, Moulvibazaar DH, and Hobiganj DH. Based on the latest census and local knowledge, in 2000 we mapped communities within a five kilometre radius of each hospital. Using random number tables, we selected one urban community within a kilometre of each hospital and one peri-urban community between one and five kilometres of each hospital. In each community, field teams interviewed about 120 contiguous households, radiating from a randomly selected starting point. We revisited the same communities in the 2003 survey.

For each district hospital, the field team interviewed consecutive outpatients as they were leaving the hospital. In the medical college hospital the teams interviewed 100 outpatients from different medical and surgical specialities in proportion to their client load. The inpatient sample was stratified into male and female wards, and into different specialities.

### Instruments and data collection

We designed the data collection instruments in consultation with the team implementing the HII programme, aiming to collect information about aspects of patient experiences and satisfaction with the hospitals that would reflect the different elements of the HII programme intervention. Table 1 shows the elements of the programme and the aspects of patient experience we linked to each aspect, recognizing there would be considerable overlap.

**Table 1:** Elements of the HII programme and indicators from the patient surveys reflecting these elements

HII programme elements	Indicators in the surveys of hospital outpatients and inpatients
Management training for senior staff	- Waiting and consultation time - Use of an agent to secure admission - Availability of medicines as reported by the patients
Behavioural change training for hospital staff	- Waiting and consultation time - Privacy arrangements (observed) - Patients' satisfaction with privacy - Patients' satisfaction with staff
Improved waste disposal arrangements	- Cleanliness (observed) - Presence of colour coded bins with instructions for use (observed) - Patients' satisfaction with cleanliness
Structural changes to improve privacy	- Patients' satisfaction with privacy - Privacy arrangements (observed)
Improved procurement systems	- Availability of medicines as reported by the patients

The *household questionnaire* enquired about the demographics and socioeconomic status of the households, and their opinion and use of the DH or MCH.

The *questionnaires for hospital outpatients and inpatients* asked about age, sex, and socioeconomic status, and included questions about use of an agent (to secure admission), waiting and consultation time, availability of medicines, and payments for aspects of their care. It asked patients about their satisfaction with aspects of their care.

Using a standardised *institutional review* proforma, the field teams recorded their observations of aspects such as cleanliness and privacy arrangements in the hospitals. We developed the instruments in English, and then translated them into Bangla, with back translation to check preservation of meaning. We piloted all the instruments to check for interpretation and flow.

For each survey we recruited and trained two teams of interviewers from Sylhet town. All supervisors and many of the interviewers from the 2000 baseline survey also participated in the 2003 survey. We contacted the hospitals ahead of each survey to secure their permission to proceed, but as far as possible we did not inform them in advance of the actual day when the team would visit to conduct the surveys.

After preliminary analysis of the surveys at each time point, we developed a focus group guide to share and discuss the main findings from the surveys, and to explore the views of the participants about potential solutions for the problems identified. Field teams returned to the 10 community sites (two for each hospital) and held separate male and female focus group discussions: 20 focus groups in all. A facilitator led each discussion and a reporter took detailed notes, later preparing a report. A small group from the research team reviewed the reports from the focus groups to identify common themes arising around the different topics discussed and to extract illustrative quotes.

### Analysis

Data entry operators used Epi-Info to enter data twice with validation to eliminate keystroke errors. Further checks sought logical inconsistencies and out of range responses. Analysis relied on CIETmap open-source software [40] which incorporates an interface to the widely used public domain R statistical software [41].

We calculated the proportions of household respondents and of hospital outpatients and inpatients with different opinions and experience of services, both in the 2000 survey and in the 2003 survey. We tested the significance of changes between the 2000 and 2003 surveys using the Mantel Haenszel procedure [42]. Due to important heterogeneity between hospitals in the changes between surveys, we tested for time changes in each hospital separately. For outcomes that changed significantly between the two surveys in bivariate analysis, we constructed multivariate models to examine whether this change over time could be explained by other variables related to the outcome. We began with saturated models including all the measured variables we believed a priori to be potentially related to the outcome and stepped down to final models that included only variables that remained significantly associated with the outcome. In addition to the time variable, the initial models included age, sex and literacy of the respondent and household head, household size, economic status indicated by the building structure (concrete and blocks, or mud/thatch) and poverty status (monthly household income Taka 5000 (US$ 83 in 2003) or less), and sex of the service provider. Associations are expressed as the adjusted OR (ORa) and 99% confidence interval (CI). We required the 99% CI in order to allow for multiple significance testing.

The research was approved by the CIETinternational ethical review panel.

## Results

### The sample populations

Across the five hospitals, in 2000 we interviewed 294 outpatients and 309 inpatients; in 2003 we interviewed 311 outpatients and 294 inpatients. Respondents from 1149 households participated in the baseline and 1230 households in the follow-up survey.

Just under half the inpatients and outpatients were female in both surveys, less than half were literate, and nearly all were from households with a monthly income of Taka 5000 (US$83 in 2003) or less (Table 2). About half the inpatients and nearly all the outpatients were from the town where the hospital was located. Most of the households surveyed were male-headed, just over half the household heads were literate, three quarters of the household respondents were women, and over two thirds had a monthly income of Taka 5000 (US$ 83) or less. About half the households had a roof constructed of mud or thatch (indicating poverty) (Table 3).

**Table 2:** Characteristics of inpatients and outpatients in the five hospitals in 2000 and 2003

	Percentage (fraction)			
Characteristic	Inpatients		Outpatients	
	2000	2003	2000	2003
Number of respondents	309	294	294	311
Female	39 (119/309)	45 (131/294)	48 (141/292)	51 (157/311)
Literate	45 (140/309)	39 (107/278)	38 (113/292)	42 (122/292)
Household monthly income Taka 5000 or less	90 (275/307)	89 (261/294)	93 (269/290)	86 (265/310)
From same town as hospital	51 (156/306)	58 (171/294)	86 (251/293)	88 (272/311)

**Table 3:** Characteristics of households in the survey in 2000 and 2003

Sample characteristic	Percentage (fraction)	
	2000	2003
Number of households	1149	1230
Head male	81 (926/1149)	93 (1146/1230)
Head literate	57 (655/1147)	58 (714/1229)
Respondent female	79 (906/1149)	76 (931/1229)
Household monthly income Taka 5000 or less	71 (811/1145)	77 (947/1229)
Constructed with mud and/or thatched roof	54 (622/1148)	46 (566/1229)

### Views and experience of hospital outpatients

Table 4 summarizes the results from the multivariate analyses of changes in outpatients' experience and views between 2000 and 2003. An outpatient was more likely to be seen within 10 minutes in 2003 than in 2000, in Sonamganj and Moulavibazar DH, and in these same hospitals an outpatient was more likely to report being seen by the doctor for at least five minutes in 2003 than in 2000. Deterioration in medicine availability was significant in three of the DH but not in the MCH. In Hobiganj DH, an outpatient was more likely to receive all prescribed medicines in 2003 than in 2000.

**Table 4:** Changes over time in outpatient experience and satisfaction

Hospital	Percentage (fraction)		Adjusted Odds Ratio	99% confidence interval
	2000	2003		
**Waited less than 10 minutes to see the doctor**				
Medical College	32 (30/93)	34 (34/100)	1.08*	0.49 – 2.56*
Sylhet DH	27 (13/48)	53 (26/49)	2.84	0.93 – 1.68
Sonamganj DH	14 (7/50)	73 (40/55)	**14.33**	**4.01 – 51.23**
Moulvibazaar DH	40 (20/52)	68 (38/56)	**7.76**	**2.05 – 29.38**
Hobiganj DH	82 (42/51)	59 (30/51)	0.53	0.15 – 1.88
**Doctor consulted for 5 minutes or more**				
Medical College	47 (42/90)	65 (64/99)	2.09*	0.97 – 4.49*
Sylhet DH	33 (16/48)	33 (16/49)	0.97*	0.32 – 2.97*
Sonamganj DH	8 (4/50)	55 (30/55)	**10.40**	**2.51 – 43.10**
Moulvibazaar DH	12 (6/52)	43 (24/56)	**5.25**	**1.30 – 21.19**
Hobiganj DH	16 (8/51)	10 (5/51)	0.58*	0.12 – 2.79*
**Got all prescribed medicines from the hospital**				
Medical College	51 (44/86)	48 (47/99)	0.86*	0.40 – 1.85*
Sylhet DH	77 (37/48)	35 (17/49)	**0.09**	**0.02 – 0.43**
Sonamganj DH	80 (39/49)	33 (17/52)	**0.11**	**0.03 – 0.39**
Moulvibazaar DH	68 (34/50)	20 (11/55)	**0.13**	**0.04 – 0.40**
Hobiganj DH	53 (27/51)	71 (35/49)	2.22*	0.75 – 6.58*
**Satisfaction with doctor**				
Medical College	68 (62/89)	70 (70/100)	1.02*	0.45 – 2.31*
Sylhet DH	63 (30/48)	73 (36/49)	1.66*	0.53 – 5.17*
Sonamganj DH	38 (19/50)	62 (34/55)	2.64*	0.94 – 7.41*
Moulvibazaar DH	45 (23/51)	68 (38/56)	2.57*	0.92 – 7.19*
Hobiganj DH	67 (34/51)	80 (41/51)	2.05*	0.63 – 6.69*
**Satisfaction with cleanliness**				
Medical College	84 (76/90)	96 (96/100)	4.11	0.97 – 17.42
Sylhet DH	83 (40/48)	100 (49/49)	-	-
Sonamganj DH	66 (40/48)	80 (44/55)	2.06*	0.65 – 6.54*
Moulvibazaar DH	77 (39/51)	79 (44/56)	1.13*	0.34 – 3.75*
Hobiganj DH	100 (51/51)	96 (49/51)	-	-
**Satisfaction with privacy**				
Medical College	67 (60/90)	75 (75/100)	1.50*	0.66 – 3.43*
Sylhet DH	71 (34/48)	92 (45/49)	**27.52**	**2.05 – 369.22**
Sonamganj DH	32 (16/50)	61 (33/54)	3.05	0.98 – 9.52
Moulvibazaar DH	49 (25/51)	55 (31/56)	1.29*	0.47 – 3.52*
Hobiganj DH	69 (34/49)	75 (38/51)	1.29*	0.41 – 4.09*
**Overall satisfaction with service**				
Medical College	52 (46/89)	70 (70/100)	2.18*	1.00 – 4.76*
Sylhet DH	52 (25/48)	69 (34/49)	2.09*	0.70 – 6.20*
Sonamganj DH	30 (15/50)	46 (25/55)	1.94*	0.68 – 5.59*
Moulvibazaar DH	44 (22/51)	34 (19/56)	0.68*	0.24 – 1.90*
Hobiganj DH	46 (23/50)	78 (39/50)	**4.13**	**1.30 – 13.07**

*Unadjusted OR and 99% CI from bivariate analysis not significant so multivariate analysis not undertaken

Figures in **bold type** indicate a significant difference that remained after multivariate analysis

Although the satisfaction of outpatients appeared slightly higher in 2003 than in 2000 (Table 4), for most aspects of satisfaction the changes over time were not statistically significant. An outpatient in Sylhet DH was more likely to be satisfied with privacy in 2003; and outpatients in Hobiganj DH were more likely to be satisfied overall with the service in 2003.

### Views and experience of hospital inpatients

Table 5 summarizes the results from the multivariate analyses of changes in inpatients' experience and views between 2000 and 2003. The proportion of inpatients who reported they secured admission without the help of an agent was higher in 2003 than in 2000 in all the hospitals. Nonetheless, the increase was significant only in Sylhet and Hobiganj DH (and could not be tested in Moulvibazaar DH). There was a tendency for satisfaction with both doctors and nurses to increase over time, but this was statistically significant only for satisfaction with doctors in the MCH. Similarly, increased satisfaction with cleanliness and overall service was only statistically significant in the MCH.

**Table 5:** Changes over time in inpatient experience and satisfaction

Hospital	Percentage (fraction)		Adjusted Odds Ratio	99% confidence interval
	2000	2003		
**Getting admission without help of any external agent**				
Medical College	73% (75/103)	88% (87/99)	2.49	0.91 – 6.85
Sylhet DH	60% (29/48)	95% (36/38)	**12.14**	**2.09 – 70.63**
Sonamganj DH	84% (45/53)	94% (48/51)	2.84*	0.48 – 16.73*
Moulvibazaar DH	87% (45/52)	**100% (56/56)**	-	-
Hobiganj DH	40% (18/45)	92% (45/49)	**33.86**	**6.59 – 173.98**
**Attended by a doctor within one hour of admission**				
Medical College	55% (56/101)	72% (69/96)	2.05*	0.94 – 4.46*
Sylhet DH	52% (25/48)	63% (24/38)	1.58*	0.50 – 4.96*
Sonamganj DH	43% (23/53)	67% (34/51)	2.61*	0.92 – 7.39*
Moulvibazaar DH	36% (18/50)	55% (31/56)	2.20*	0.79 – 6.15*
Hobiganj DH	45% (23/51)	29% (14/48)	0.50*	0.17 – 1.49*
**No more than one attendant with the patient**				
Medical College	54% (56/104)	52% (51/99)	0.91*	0.44 – 1.88*
Sylhet DH	64% (30/47)	82% (31/38)	2.51*	0.67 – 9.39*
Sonamganj DH	43% (23/53)	47% (24/51)	1.16*	0.42 – 3.22*
Moulvibazaar DH	46% (24/52)	49% (27/55)	1.13*	0.41 – 3.07*
Hobiganj DH	48% (25/52)	60% (30/50)	1.62*	0.58 – 4.56*
**Satisfaction with doctor**				
Medical College	63% (64/102)	83% (82/99)	**3.29**	**1.33 – 8.14**
Sylhet DH	92% (43/47)	90% (34/38)	0.79*	0.12 – 5.41*
Sonamganj DH	56% (27/48)	77% (39/51)	2.53*	0.82 – 7.79*
Moulvibazaar DH	56% (29/52)	68% (38/56)	1.67*	0.60 – 4.70*
Hobiganj DH	65% (34/52)	76% (38/50)	1.68*	0.54 – 5.23*
**Satisfaction with nurse**				
Medical College	64% (66/104)	74% (73/99)	1.62*	0.74 – 3.55*
Sylhet DH	87% (41/47)	97% (37/38)	5.41*	0.41 – 72.19*
Sonamganj DH	76% (40/53)	80% (41/51)	1.33*	0.39 – 4.56*
Moulvibazaar DH	62% (32/52)	70% (39/56)	1.43*	0.50 – 4.10*
Hobiganj DH	77% (40/52)	92% (46/50)	3.45*	0.75 – 15.96*
**Satisfaction with cleanliness**				
Medical College	38% (39/104)	59% (58/99)	**2.46**	**1.17 – 2.15**
Sylhet DH	92% (43/47)	100% (38/38)	-	-
Sonamganj DH	43% (23/53)	61% (31/51)	2.02*	0.72 – 5.65*
Moulvibazaar DH	25% (13/52)	45% (25/56)	2.42*	0.83 – 7.06*
Hobiganj DH	77% (39/51)	56% (28/50)	0.39*	0.13 – 1.19*
**Satisfaction with privacy**				
Medical College	39% (40/103)	47% (46/98)	1.39*	0.67 – 2.91*
Sylhet DH	69% (31/45)	58% (22/38)	0.62*	0.19 – 2.04*
Sonamganj DH	36% (19/53)	39% (20/51)	1.15*	0.40 – 3.30*
Moulvibazaar DH	44% (23/52)	23% (13/56)	0.38*	0.13 – 1.12*
Hobiganj DH	42% (22/52)	58% (29/50)	1.88*	0.67 – 5.30*
**Satisfaction with overall service**				
Medical College	40% (41/103)	63% (62/98)	**2.54**	**1.20 – 5.36**
Sylhet DH	81% (35/43)	89% (34/38)	1.94*	0.36 – 10.48*
Sonamganj DH	42% (22/52)	51% (26/51)	1.42*	0.51 – 3.95*
Moulvibazaar DH	26% (12/47)	36% (20/56)	1.62*	0.53 – 4.98*
Hobiganj DH	49% (25/51)	62% (31/50)	1.70*	0.60 – 4.82*

*Unadjusted OR and 99% CI from bivariate analysis not significant so multivariate analysis not undertaken

Figures in **bold type** indicate a significant difference that remained after multivariate analysis

### Observations in the hospitals

Using the same direct observation protocol, field teams recorded improvement in general cleanliness in all the hospitals in 2003 compared with 2000. In 2003 the field teams observed new colour-coded waste bins in outpatient areas and on the wards, with posters to explain their use. Except in Hobiganj DH, where there was an improvement, the toilets were just as dirty in 2003 as in 2000. In both 2000 and 2003 the teams recorded that outpatients were examined in an open room with other patients and their relatives also present. Only in Sylhet DH was there a screen in some consulting rooms providing privacy for examinations. On the wards there were no curtains between the beds or any portable screens to allow privacy when examining patients. In 2003 the teams noted some new brick partitions between groups of beds in three of the DH but no screening for individual beds.

### Household views and use of hospitals

Table 6 summarizes the multivariate analyses of household views and use of the hospitals during the year before each survey. Households in the catchment area of each of the hospitals were significantly more likely to rate their DH as good in 2003 than in 2000. The proportion of households using the hospitals for outpatient care did not change significantly between 2000 and 2003. Except for Sylhet DH, more households apparently used the intervention hospitals for inpatient care in 2003 than in 2000, but the increase was not statistically significant.

**Table 6:** Changes over time in household perceptions and use of their district hospital and medical college hospital

Hospital	Percentage (fraction)		Adjusted Odds Ratio	99% confidence interval
	2000	2003		
**Consider the hospital to be good or very good**				
Medical College	28% (140/496)	37% (178/479)	1.51	0.73 – 3.14
Sylhet DH	26% (128/494)	40% (167/416)	**1.87**	**1.29 – 10.42**
Sonamganj DH	9% (17/201)	23% (57/248)	**3.39**	**2.36 – 4.88**
Moulvibazaar DH	14% (28/206)	26% (65/253)	**2.06**	**1.47 – 3.30**
Hobiganj DH	26% (64/246)	42% (103/244)	**2.14**	**1.38 – 3.30**
**Used hospital for outpatient care during the year prior to survey**				
Medical College	33% (164/496)	34% (164/479)	1.05*	0.74 – 1.51*
Sylhet DH	5% (25/496)	6% (28/479)	1.28*	0.61 – 2.68*
Sonamganj DH	31% (62/201)	41% (102/247)	1.58*	0.94 – 2.64*
Moulvibazaar DH	20% (42/206)	26% (67/254)	1.40*	0.79 – 2.49*
Hobiganj DH	16% (39/245)	18% (44/244)	1.16*	0.62 – 2.16*
**Used hospital for inpatient care during the year prior to survey**				
Medical College	9% (46/494)	14% (66/479)	1.56	0.92 – 2.62*
Sylhet DH	1% (7/496)	1% (5/479)	0.74*	0.16 – 3.34*
Sonamganj DH	9% (18/201)	15% (36/247)	1.73*	0.79 – 3.79*
Moulvibazaar DH	4% (9/206)	8% (21/254)	1.97*	0.70 – 5.58*
Hobiganj DH	8% (20/244)	10% (23/243)	1.17*	0.51 – 2.67*

*Unadjusted OR and 99% CI from bivariate analysis not significant so multivariate analysis not undertaken

Figures in **bold type** indicate a significant difference that remained after multivariate analysis

### Community views and suggestions

Focus group participants were not surprised by the results from the surveys. When considering ways of improving cleanliness, most felt that hospital management should be responsible for improving cleanliness, for example, through employing and training more cleaning staff. However, they recognized that patients also had a responsibility to help keep hospitals clean, for example by not spitting pan, not dropping waste in and around the premises, using waste bins where provided, and not bringing too many attendants with them. Despite the intention of the HII programme to improve privacy, this was not apparently achieved. Both male and female groups were concerned about continuing lack of privacy in the hospitals. In order to improve privacy, particularly for female patients, they suggested that men should not be allowed to enter female wards or use female toilets. Presented with the information about the high number of attendants with patients, they recognized that the presence of several attendants for each patient hampered the work of the hospital staff, and posed hygiene and privacy problems, but they pointed out that patients needed the attendants because they did not get adequate personal care from the hospital staff.

## Discussion

In some of the hospitals, there was some improvement in patients’ experience with administrative aspects of service quality such as shorter waiting times, longer consultations with the doctor, and not requiring the support of external agents to secure admission. There were also some improvements in patients’ satisfaction with cleanliness and with staff. The improvements were patchy and did not occur in all the hospitals. No one hospital had improvements in all aspects measured. The MCH did relatively well, especially for inpatient care, while in Hobiganj DH some aspects of patient experience apparently deteriorated. Public opinion about the hospitals in their catchment areas improved over the period of the HII.

A limitation of our study is that we did not compare the HII programme hospitals with hospitals not in the programme and therefore we cannot confidently attribute to the HII any changes we detected. Improvements related to these hospitals may have been part of an overall improvement in health services, perhaps as a result of the overall HPSP that was taking place in the same period. However, there is no evidence of such an overall improvement in service delivery from government hospitals. A study published in 2007 found that patients in Bangladesh rated public (government) hospitals less positively than private hospitals [38]. Three large national surveys examined household views, use and experience of health services over the period of the HPSP [6,43,44]. Over the period, household use of government health services, including hospitals, fell, and the opinion of households and service users about government health services deteriorated [45]. Our findings of increased use of the hospitals (although non-significant) and improvements in some aspects of patient experience in the hospitals run counter to the prevailing trends; this strengthens the argument they may be related to the HII programme.

We asked patients about their satisfaction with the care they received. Patient satisfaction ratings can be importantly affected by factors such as culture, expectations, and sense of entitlement [16,46,47]. However, since we revisited the same communities and hospitals in the two surveys, it is unlikely that big shifts in these factors explained the differences we found over time. Allowing patients to judge and evaluate care using their own standards empowers the patient and fosters patient centred care [48]. Ratings may be prone to errors if there is a delay between experience and data collection [49], so most studies rely on contacting patients at the health facility [50]. However, responses to questions on general opinion and service expectations may be biased by the actual service received [51] and consulting only service users excludes the views of people who do not use the services even though they may need them [13].

An important aspect of service delivery deteriorated in most of the hospitals during the HII programme; in 2003 outpatients were less likely than in 2000 to report they received all the prescribed medicines from the hospital. National surveys over the same period reported a significant fall in the proportion of users of government health facilities who received all the required medicines, and non-availability of medicines was associated with lower patient satisfaction [45]. There was also no reduction in the high proportion of inpatients (half or more) with two or more attendants staying with them. The HII apparently did not effectively tackle these problems. Other reports have noted shortcomings of the HII programme. A World Bank report in 2005 concluded there had been little progress towards meaningful autonomy and local accountability of managers in the HII pilot hospitals (which by then included others in addition to the five we studied) and noted the overall lack of benchmarks for evaluating the programmes [52]. Another report noted lack of effectiveness of the Health Advisory Committees set up under the HII programmes to include the community voice in management of the hospitals [53].

Other studies in Bangladesh suggest that even if problems of access could be resolved, quality factors would still influence patients’ use of services [54]. Increased dissatisfaction of the public and service users is accompanied by decreasing use of government health facilities and an increase in the unmet need for health care, especially among the more vulnerable [45-55].

## Conclusion

The social audit provided useful information on the effects of the HII programme from the perspective of service users and households in the hospital catchment areas. Some improvements occurred in the HII hospitals over the period of the programme, in the face of general deterioration in views and experience of government health services nationally over the same period. The programme failed to prevent the general reduction in availability of medicines in government facilities that occurred over the period. Repeated assessments of patients' ratings of care in hospitals together with documentation of potential users’ view and use of hospital services could be a useful part of efforts to improve the quality of hospital services in Bangladesh.

## List of abbreviations used

CI: Confidence Interval; DH: District Hospital; HII: Hospital Improvement Initiative; HPSP: Health and Population Sector Programme; MCH: Medical College Hospital; ORa: Adjusted Odds Ratio

## Competing interests

The authors declare they have no competing interests.

## Authors' contributions

KO contributed to the design of the study, led the data collection, undertook data analysis, and drafted the paper. AC contributed to the design, supported data analysis, and helped to draft the paper. NA designed the methods used, supported data analysis, and helped to draft the paper.
